# Separation-Individuation in Patterns in Turkey: An Investigation of Developmental, Gender and Contextual Differences

**DOI:** 10.1192/j.eurpsy.2023.1570

**Published:** 2023-07-19

**Authors:** T. Bildik, B. Şentürk Pilan, B. Özbaran, Z. Yüncü, S. Köse

**Affiliations:** Child and Adolescent Psychiatry, Ege University Medical School, Izmir, Türkiye

## Abstract

**Introduction:**

There is still a *paucity* of *studies* exploring the impacts of teenage employment on mental health and development. In many developing countries, youths participate intensively in the teenage labor force. The involvement of adolescent in the world of work would promote the development of responsibility and prepare the young person to function more effectively in adult roles.

**Objectives:**

The purposes of the present study were to examine the adolescent separation-individuation among an understudied and vulnerable population; and to examine the Separation-Individuation Test of Adolescence (SITA) for revealing the differentiation working male group from student male group.

**Methods:**

Participants completed the SITA. The group of study 1 consisted of 618 high school and 252 university students between the ages of 14 and 24, 489 of which were girls and 381 were boys. In Study 2, participants were 269 male adolescents between the ages of 15 and 17, 141 of which were full-time worker and 128 high school students. In study 1, these results were consistent with the r*approchement subphase* of the separation-*individuation process in adolescence -*the striking desires for intimacy and independence.

**Results:**

Our results support the gender specific patterns of identity formation and the argument that connectedness in close relationships plays a central role in the female identity development. Interestingly, there were no significant gender differences in the tendency to view close interpersonal relationships as threatening to their sense of independence and selfhood, and to deny or avoid dependency needs. The reason for this might be the cultural differences between the individualistic and collectivistic cultures. In study 2, working males could be distinguished from student counterparts by their increased tendency to seek close interpersonal ties with caretakers. The finding also suggested that student males reflected characteristics of the traditional view of adolescents’ independence striving. Across both studies it was found that the traditional *Turkish child-rearing patterns* have been characterized by an emphasis on interpersonal relationships and close ties with family than individualistic cultures

**Conclusions:**

Our results revealed that the subscales of SITA –especially dependency denial, rejection expectancy, nurturance seeking, engulfment anxiety, and teacher enmeshment- can help clinicians for assessing the adolescent separation-individuation process in a reliable way. We suggested that adolescent employment would lead to the *difficulties* in developing bonds with parents because of the adolescents’ long work hours, the early involvement with the world of work would lead to the *withdrawal from the more dependent, preadultlike student role, and* student adolescents would reflect characteristics of the traditional view of adolescents’ independence striving.

**Disclosure of Interest:**

None Declared

